# Identifying Patterns of Late Effects With Latent Class Analysis Among Adolescent and Young Adult Thyroid Cancer Survivors in California and Utah

**DOI:** 10.1002/cam4.71316

**Published:** 2025-11-27

**Authors:** Judy Y. Ou, Theresa Keegan, Qian W. Li, Heydon K. Kaddas, Renata Abrahão, Ann Brunson, Candice A. M. Sauder, Jessica Chubak, Lawrence H. Kushi, Erin E. Hahn, Chun Chao, Hazel B. Nichols, Anne C. Kirchhoff

**Affiliations:** ^1^ Huntsman Cancer Institute at the University of Utah Salt Lake City Utah USA; ^2^ Department of Pediatrics University of Utah Salt Lake City Utah USA; ^3^ University of California, Davis Comprehensive Cancer Center Sacramento California USA; ^4^ Kaiser Permanente Washington Health Research Institute Seattle Washington USA; ^5^ Kaiser Permanente Northern California Division of Research Pleasanton California USA; ^6^ Department of Research and Evaluation Kaiser Permanente Southern California Pasadena California USA; ^7^ Department of Health Systems Science Kaiser Permanente Bernard J. Tyson School of Medicine Pasadena California USA; ^8^ Department of Epidemiology University of North Carolina Gillings School of Global Public Health Chapel Hill North Carolina USA

**Keywords:** adolescent, late effects, thyroid

## Abstract

**Introduction:**

Thyroid cancer is one of the most common cancers in adolescents and young adults (AYA, 15 to 39 years), with an excellent 5‐year survival of 98%. However, treatments for thyroid cancer such as radioactive iodine and thyroid hormone suppression may increase the risk for multiple late effects (LEs). We investigated the incidence of severe LE that clustered in AYA thyroid cancer survivors in a large population‐based cohort.

**Methods:**

California and Utah Cancer Registry records identified AYAs diagnosed with first thyroid cancer during 2006–2018 linked to statewide hospitalization, ambulatory surgery, and emergency department data. Cohort entry began 2 years from diagnosis. Severe LE included cardiovascular, respiratory, renal, and liver diseases, diabetes, and second cancers. Cumulative incidence of each LE, accounting for the competing risk of death, was calculated. Latent class analysis (LCA) identified clustering of LE over the study period. The number of LE classes was identified by selecting models with the lowest likelihood‐ratio G^2^ statistic, Akaike's Information Criterion, and Bayesian Information Criterion. Probabilities of each LE are presented in each class.

**Results:**

Of 14,268 survivors, median follow‐up time was 7 years. The LCA model identified 3 classes: 88% with low LEs, 9% experiencing moderate LEs with elevated probability of diabetes, liver, and respiratory conditions, and the remaining 3% experiencing the highest probability of all LEs, including cardiovascular disease. Non‐Hispanic (nH)‐Black and Hispanic survivors, those on public insurance, residing in lower socioeconomic status neighborhoods, or diagnosed with distant stage disease experience greater odds of being in the moderate and cardiovascular classes.

**Conclusion:**

While most survivors of AYA thyroid cancer have a low incidence of LEs, a small proportion have a high probability of multiple morbidities. Multidisciplinary survivorship care should include identifying and supporting thyroid cancer survivors at higher risk for developing multiple LEs through early screening.

## 1 Introduction

Thyroid cancer is one of the most common cancers among adolescents and young adults (AYA) aged 15 to 39 years [1]. Because of their young age and effective treatment, AYAs with thyroid cancer have low risk for mortality as five‐year survival is 98.1% [1]. Patients are treated with thyroidectomy, as well as adjuvant radioactive iodine therapy and thyroid hormone suppression therapy [2]. Both radioactive iodine therapy and thyroid hormone suppression impair thyroid function and are hypothesized to accelerate the aging process [3, 4], increasing the risk for chronic conditions usually found in older populations like diabetes, cardiovascular disease, and pulmonary fibrosis [3, 5, 6, 7]. Thyroid hormone suppressive therapy can lead to cardiac dysfunction such as increased heart rate and decreased stroke volume [8, 9]. Chronic health conditions related to thyroid cancer and its treatments (late effects) can arise starting a few months to years after initial thyroid cancer therapy. As AYA thyroid cancer survivors may live for decades beyond treatment completion, their lifetime potential for developing a greater number of late effects is higher relative to persons without a cancer history [4, 10, 11, 12].

While thyroid cancer patients have favorable outcomes, the potential for late effects for those diagnosed at younger ages is apparent. In an analysis of Utah's statewide electronic health records, AYA thyroid cancer survivors first diagnosed at age < 40 had an increased risk for hypertension and cardiomyopathy within 5 years of diagnosis relative to an age‐matched population sample [4, 13]. A statewide analysis using cancer registry and hospitalization data in California found that the incidence of late effects was higher among AYAs with thyroid cancer of Black or Hispanic race/ethnicity, those residing in lower socioeconomic neighborhoods, and those with public insurance or no insurance [14]. How late effects cluster among AYA thyroid cancer survivors, however, may be masked by the methods used in previous surveillance papers that considered each late effect as a separate outcome.

Identifying patterns of late effects among AYA thyroid cancer survivors is critical because multimorbidity can deteriorate survivors' quality of life, educational attainment, and employment opportunities [15, 16]. Here we report on findings from the “Valuing Opinions and Insight from Cancer Experiences” (VOICE) study [17], to explore patterns of late effects among AYA thyroid cancer survivors that require medical care in inpatient hospitalization, emergency department, or ambulatory surgery settings. Our goal was to identify patterns of late effects and to determine whether subgroups of AYA survivors are at risk for late effect clusters identified with latent class analysis.

## 2 Materials and Methods

### 2.1 “Valuing Opinions and Insight From Cancer Experiences” (VOICE) Data Overview

We combined data from the California Cancer Registry (CCR) and the Utah Population Database (UPDB)/Utah Cancer Registry (UCR). CCR is linked to the California Department of Health Care Access and Information (HCAI) data on hospitalizations, emergency room visits, and ambulatory surgery visits for the entire state. UPDB is a unique resource that links to statewide administrative records for the entire Utah population to cancer diagnosis and treatment information available from the UCR. Like the CCR, UPDB is linked to statewide data on hospitalization, emergency room visits, and ambulatory surgery visits. For both states, statewide inpatient hospitalization data includes a record for each inpatient discharged from any non‐federal acute care hospital. This study was approved by the Institutional Review Boards at the University of Utah, the Utah Resource for Genetic and Epidemiologic Research, the California Committee for the Protection of Human Subjects, and Kaiser Permanente Northern California Institutional Review Board.

### 2.2 Cohort Selection and Follow‐Up

Both UCR and CCR are members of the Surveillance, Epidemiology, and End Results (SEER) program that tracks cancer diagnosis and treatment information for all persons in their state of residence. We identified thyroid cancer survivors diagnosed between ages 15 and 39 years from 2006 to 2018 in California and Utah who were alive and had > 2 years of follow‐up after diagnosis. Survivors were followed from 2 years after thyroid cancer diagnosis to death, the date of last follow‐up, or the end of the study on December 31, 2020, whichever occurred first.

### 2.3 Outcome Ascertainment

Late effects were defined as diseases that first occurred starting ≥ 2 years after the thyroid cancer diagnosis. The exception to this is second cancers, which could be diagnosed 60 days after the diagnosis of thyroid cancer, as done in prior studies of second cancers [18]. We included common late effect disease categories in the study: cardiovascular, diabetes Type 1 or 2, liver, respiratory, renal, and second cancers. We reviewed all hospitalization, emergency room visits, and ambulatory surgery visits data for the International Classification of Diseases, Ninth or Tenth Revision, Clinical Modification (ICD‐9‐CM/ICD‐10‐CM) and Current Procedure Terminology (CPT) codes of interest and identified individuals with at least one ICD‐9/ICD‐10 code that fell into each disease category (Yes/No). Second cancers were defined as tumors that were diagnosed ≥ 60 days after the first primary thyroid cancer using the SEER data. Second primary malignant neoplasms with histologic characteristics similar to those of their corresponding primary tumor were reviewed to verify that no second cancers would be considered recurrences according to the updated SEER multiple primary coding rules or best clinical practice [19]. Pre‐existing late effects identified among AYAs in the five years prior to cancer diagnosis were not included as outcomes in our analyses.

### 2.4 Demographic, Cancer Diagnosis, and Treatment Data

Sex, race/ethnicity, age at diagnosis, year of diagnosis, stage at diagnosis (local, regional, distant), vital status and year of death, and health insurance status at first cancer diagnosis were provided by the statewide cancer registries. Race/ethnicity was categorized as Non‐Hispanic White, Non‐Hispanic Black, Hispanic (any race), Asian/Pacific Islander, American Indian, and Other/Unknown. First course cancer treatment was ascertained using SEER registry data, including surgeries and radioactive iodine treatment. The SEER registry data include treatments conducted in outpatient and inpatient settings. We classified treatment as: total thyroidectomy and radioactive iodine, total thyroidectomy without radioactive iodine, partial thyroidectomy with radioactive iodine, partial thyroidectomy without radioactive iodine, and no thyroidectomy. No thyroidectomy included patients with and without radioactive iodine treatment.

Neighborhood socioeconomic status (SES) at diagnosis was ascertained via linkage between cancer registry data and Census records. The cancer registries computed the Yost index, a measure of socioeconomic status created using data on household income and employment, for each census tract documented at diagnosis [20]. Neighborhoods were classified into highest, middle, and lowest SES by tertile based on the state of residence.

### 2.5 Statistical Methods

We computed descriptive statistics for demographic characteristics. We computed cumulative incidence functions (CIF) for each late effect for the entire cohort using non‐parametric models accounting for the competing risk of death [21]. We utilized latent class analysis (LCA) to determine if the response patterns identified discrete, mutually exclusive population classes. LCA is a type of mixture modeling using categorical data [22]. The model assumes that the observed data distribution is the result of a latent mixture of underlying distributions. The LCA models describe these latent classes based on which variables follow the same distribution [22]. The model also provides posterior probabilities, which describe the degree to which a specific measured variable describes that latent class. The higher the probability, the more likely that members of that latent class will provide the same observed response pattern. Posterior probabilities 0.5–0.7 indicate moderate homogeneity while probabilities ≥ 0.8 indicate high homogeneity. We used an iterative approach to identify the number of latent classes that best fit the model according to the lowest Akaike information criterion (AIC), G^2^ value, and contextual contribution of the classes [23, 24].

We computed the LCA using late effects measured from cohort entry to the end of follow‐up (up to 15 years). Nearly everyone in the cohort was followed for 3 years after cohort entry (5 years after diagnosis); 25% were followed for > 10.7 years. Because survivors may accumulate more late effects as time from diagnosis increases [12] and had varying lengths of follow‐up, we also classified late effects occurring after 3 years of follow‐up (short‐term). Each individual was classified into one of the latent groups identified in the long‐ and short‐term LCAs. We then plotted the distributions of late effects by LCA classes to determine if the LCA classes represented distinct groups with distributions of disease (nonparallel lines) or if they represented a single group that was artificially split into multiple groups representing varying degrees of disease (parallel lines) [22]. We also created a bar plot to determine if the classes identified in the short‐term LCA overlapped with classes in the long‐term LCA; considerable overlap may imply that the patterns remained consistent over time.

We estimated the association of demographic characteristics, stage at diagnosis, and treatment with outcomes defined by the long‐term LCA classes using multinomial logistic regression models. To compare results with other standard methods, we computed risk for late effects using multinomial logistic models that examined late effects as a summed number (none (reference group), 1, and ≥ 2) and Cox proportional hazards models that examined each late effect as a separate outcome. For all analyses, age at diagnosis, sex, race/ethnicity, health insurance status at diagnosis, neighborhood SES, stage at diagnosis, and treatment were considered risk factors and included in the models. Models controlled for year of diagnosis and state of residence.

## 3 Results

**FIGURE 1:**
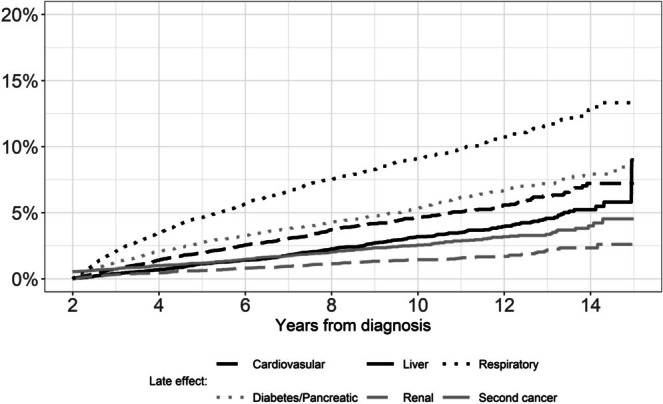
Cumulative incidence for each late effect among AYA thyroid cancer survivor cohort.

There were 14,268 AYA thyroid cancer survivors diagnosed between 2006 and 2018 followed for a median of 7.7 (interquartile range: 4.9–10.7) years (Table 1). Most cohort members were diagnosed between age 30 and 39 years (61.9%), female (83.0%), Non‐Hispanic White (49.3%), and had private or military insurance (82.4%). Local/regional tumors (97.1%) treated with total thyroidectomy with (47.2%) or without radioactive iodine (35.6%) were the most common stage and treatments. The CIF curves showed that the incidence of respiratory disease was the highest of the late effects followed by diabetes (Figure 1). The cumulative incidence of second cancers was 2% at 7 years and 4% at 14 years after diagnosis.

**TABLE 1:** Demographic and clinical description of adolescent and young adult (AYA) thyroid cancer survivor cohort, Utah and California, 2006–2018.

	Total (*N* = 14,268)	
	*n*	%
Age at diagnosis (years)		
15–19	766	5.4
20–29	4666	32.7
30–39	8836	61.9
Sex		
Female	11,840	83.0
Male	2428	17.0
Year of diagnosis		
2015–2018	4116	28.9
2012–2014	3707	26.0
2009–2011	3477	24.4
2006–2008	2968	20.8
Race/ethnicity		
Non‐Hispanic White	7029	49.3
Non‐Hispanic Black	367	2.6
Hispanic	4528	31.7
Asian/Pacific Islander	2039	14.3
American Indian	90	0.6
Other/unknown	215	1.5
Health insurance		
Private/military	11,761	82.4
Public	1993	14.0
Unknown	514	3.6
Neighborhood socioeconomic status		
Unknown	151	1.1
Lowest	3701	25.9
Middle	5307	37.2
Highest	5109	35.8
Stage at diagnosis		
Local/regional	13,860	97.1
Distant	248	1.7
Unknown	160	1.1
Treatment		
Total thyroidectomy and radioactive iodine	6734	47.2
Total thyroidectomy without radioactive iodine	5124	35.9
Partial thyroidectomy with radioactive iodine	710	5.0
Partial thyroidectomy without radioactive iodine	1544	10.8
No thyroidectomy	156	1.1
Number of late effects		
0	12,067	84.6
1	1685	11.8
2+	516	3.6
Vital status		
Alive	14,140	99.1
Dead	128	0.9

In the long‐term LCA, an estimated 88% had few late effects (“Low”), 3% were estimated to have moderately high probabilities for cardiovascular late effects (“Cardiovascular”), and the remaining estimated 9% had probabilities between 0.17 and 0.23 for diabetes, liver, and respiratory late effects (“Moderate”) (Table 2). The short‐term LCA estimated that 88% of survivors were in the Low class. The short‐term LCA also classified cardiovascular and renal late effects together with 1% of the population falling into this class (Cardiovascular and Renal). We found low but elevated probabilities for diabetes, liver, and respiratory late effects in an estimated 11% of the population in the short‐term LCA (Moderate).

**TABLE 2:** Latent class analysis (LCA) for long‐term and short‐term patterns in late effects among AYA thyroid cancer survivor cohort.

	Long‐term (cohort entry to end of follow‐up)		
	Cardiovascular	Moderate	Low
Actual cohort percentages	2.1%	4.9%	93%
Percentages estimated from LCA^a^	3%	9%	88%
Class probabilities from LCA			
Cardiovascular	0.62**	0.07	0.01
Diabetes/Pancreatic	0.37	0.23	0.01
Liver	0.21	0.17	0.00
Respiratory	0.41	0.23	0.04
Renal	0.32	0.02	0.00
Second cancer	0.10	0.05	0.01

	Short‐term (cohort entry to 3‐years^b^)		
	Cardiovascular and Renal	Moderate	Low
Actual cohort percentages	0.6%	7.2%	92.2%
Percentages estimated from LCA^a^	1%	11%	88%
Class probabilities from LCA			
Cardiovascular	0.73**	0.15	0.01
Diabetes/Pancreatic	0.48	0.22	0.01
Liver	0.23	0.15	0.00
Respiratory	0.49	0.26	0.04
Renal	0.58**	0.04	0.00
Second cancer	0.10	0.06	0.01

^a^ Calculated proportion of individuals in the data set that are predicted to belong to each identified latent class.

^b^ 3‐years after cohort entry (which starts at 2 years after diagnosis) also refers to 5 years after cancer diagnosis.

** Probabilities ≥ 0.50.

**FIGURE 2:**
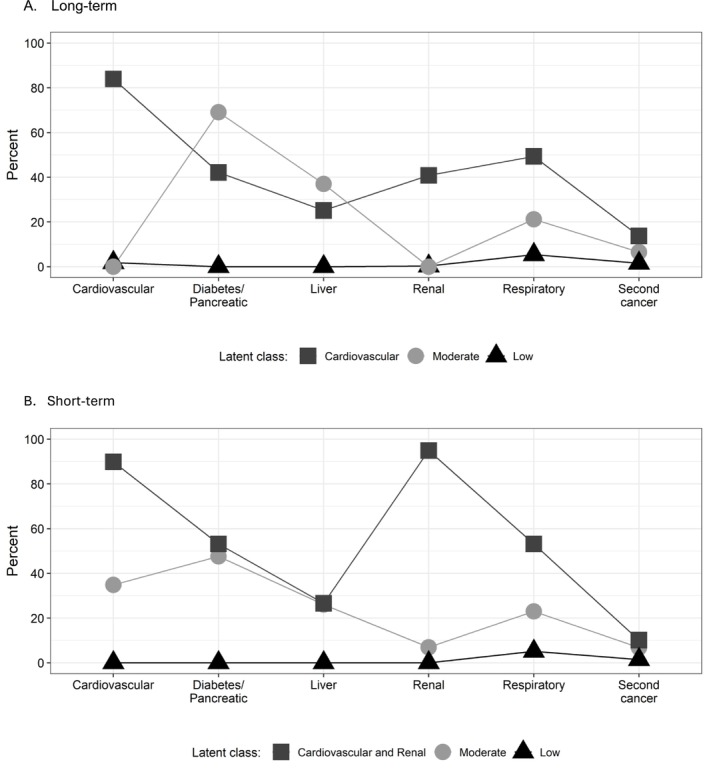
Distribution of late effects within each long‐term (A) and short‐term (B) latent class among AYA thyroid cancer survivor cohort. (A) Long‐term. (B) Short‐term. This figure shows that the latent class analysis classes represent distinct groups with distributions of disease (non‐parallel lines).

We plotted the prevalence of each late effect by the long‐term LCA and the short‐term LCA classes. The patterns in the prevalence of each late effect support that these classes represent distinct distributions rather than varying degrees of severity from a single omnibus distribution (Figure 2). In the long‐term LCA, 80% of those in the Cardiovascular class had at least one cardiovascular late effect and 40% had at least one respiratory late effect. In the Moderate class, 75% had at least one diagnosis of diabetes, over 20% had one report of liver late effects, and 20% had at least one report of respiratory late effects. In the short‐term LCA, the Cardiovascular and Renal classes had 90% and nearly 100% prevalence of each late effect, respectively. In Moderate, the prevalence of diabetes was nearly 50%, and the prevalence of respiratory disease was 20%.

**FIGURE 3:**
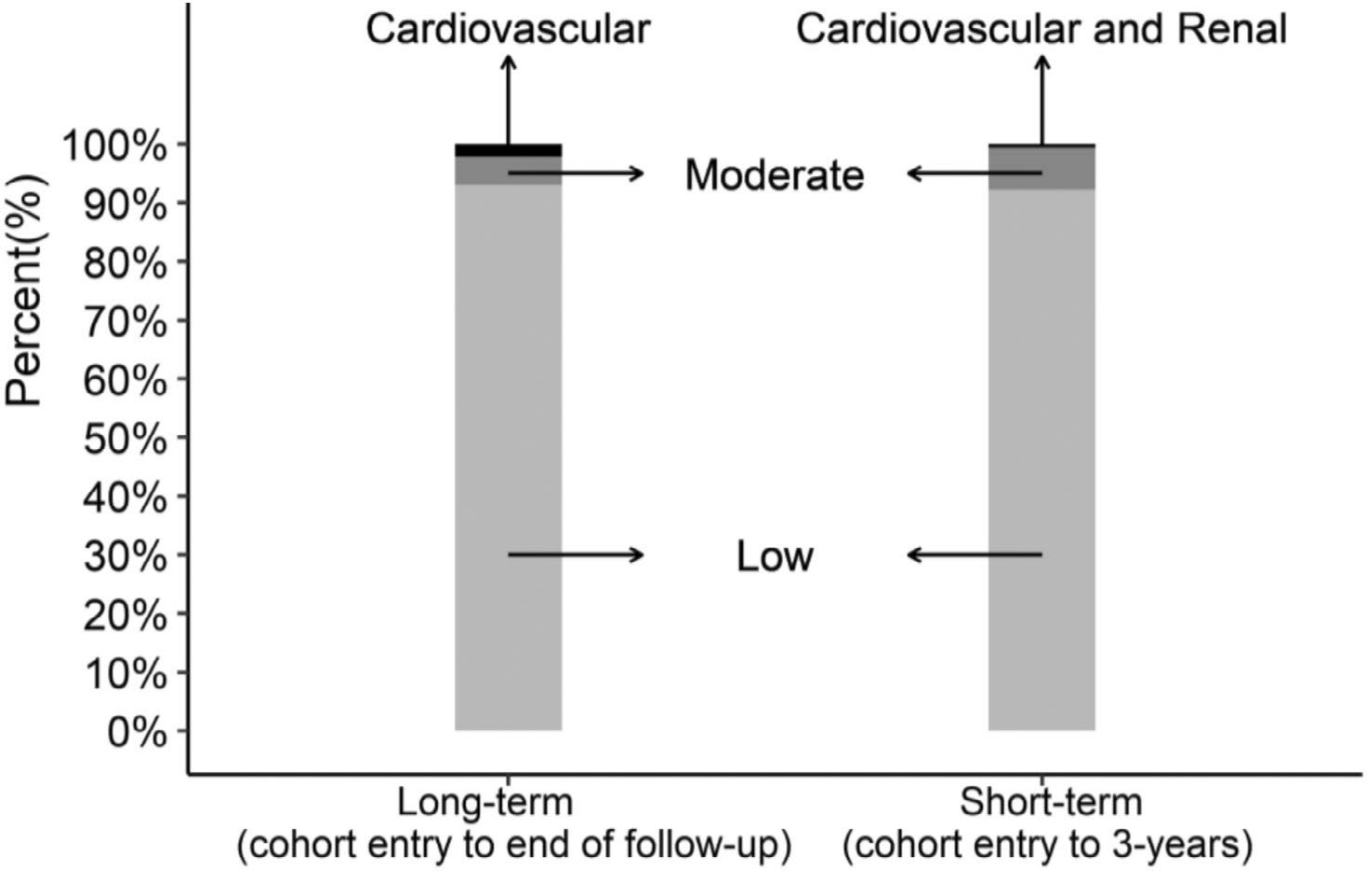
Distribution of long‐term and short‐term patterns in latent class groups among AYA thyroid cancer survivor cohort.

Figure 3 shows that the LCA model categorized nearly 90% of participants as being in the Low late effects class both in the short‐term and long‐term. A small percentage of survivors were classed as Low or Moderate in the short‐term model but were in the Cardiovascular class in the long‐term model (*n* = 199). As the long‐term Moderate class did not have any cardiovascular disease, this shows that the short‐ and long‐term classification schema may have been affected by conditions that appear due to differences in follow‐up time.

The classification of late effects in the long‐term LCA and summed number of late effects (Table 3) supported higher odds for late effects among survivors diagnosed at age 20 to 39 relative to those diagnosed between ages of 15 and 19 years, survivors with public health insurance relative to survivors with private insurance, and those living in neighborhoods with the lowest socioeconomic status relative to residence in the highest socioeconomic status for both. While there were no significant differences by sex in the LCA model, the summed number model had a significant elevation in the odds for having one late effect among female survivors (OR = 1.27, 95% CI = 1.10, 1.48; Table 3). The models also reported some differences in the odds for late effects according to race/ethnicity. The LCA model reported higher odds for Moderate among non‐Hispanic Black survivors and Hispanic survivors relative to non‐Hispanic White survivors (nH Black OR = 1.71, 95% CI = 1.07, 2.74; Hispanic OR = 2.20, 95% CI = 1.83, 2.66). The summed number model reported a significant decrease in the odds for having one late effect among Asian/Pacific Islanders (OR = 0.69, 95% CI = 0.58, 0.83) and an increase in odds for two or more late effects among non‐Hispanic (nH)‐Black survivors and Hispanic survivors (nH‐Black OR = 1.87, 95% CI = 1.18, 2.98; Hispanic OR = 1.50, 95% CI = 1.21, 1.86). Results for the short‐term LCA model results as the outcome variable are in Table S1.

**TABLE 3:** Risk factors associated with group classification identified in the long‐term latent class analysis and summed total number of late effects among AYA thyroid cancer survivor cohort.

	Latent class analysis groups		Summed total number of late effects	
	(Reference = Low)		(Reference = None)	
	Moderate	Cardiovascular	One late effect	Two or more late effects
	*N* = 692 (4.9%)	*N* = 306 (2.1%)	*N* = 1685 (11.8%)	*N* = 516 (3.6%)
	OR (95% CIs)	OR (95% CIs)	OR (95% CIs)	OR (95% CIs)
Age at diagnosis, year				
15–19	Reference	Reference	Reference	Reference
20–29	**1.59 (1.02, 2.49)**	**2.28 (1.04, 4.99)**	1.20 (0.94, 1.55)	**2.29 (1.28, 4.10)**
30–39	**2.64 (1.71, 4.07)**	**4.03 (1.87, 8.65)**	**1.49 (1.16, 1.90)**	**3.73 (2.11, 6.58)**
Sex				
Female	0.93 (0.75, 1.14)	0.70 (0.53, 0.93)	**1.27 (1.10, 1.48)**	0.83 (0.66, 1.04)
Male	Reference	Reference	Reference	Reference
Race/ethnicity				
Non‐Hispanic White	Reference	Reference	Reference	Reference
Non‐Hispanic Black	**1.71 (1.07, 2.74)**	1.45 (0.80, 2.64)	1.10 (0.80, 1.51)	**1.87 (1.18, 2.98)**
Hispanic	**2.20 (1.83, 2.66)**	1.14 (0.86, 1.50)	1.10 (0.97, 1.24)	**1.50 (1.21, 1.86)**
Asian/Pacific Islander	1.23 (0.94, 1.61)	0.81 (0.55, 1.19)	**0.69 (0.58, 0.83)**	1.03 (0.77, 1.38)
American Indian	1.51 (0.89, 2.56)	0.98 (0.42, 2.26)	1.12 (0.79, 1.59)	1.41 (0.77, 2.59)
Health insurance				
Private/military	Reference	Reference	Reference	Reference
Public	**1.81 (1.48, 2.21)**	**2.41 (1.83, 3.18)**	**1.59 (1.38, 1.83)**	**2.37 (1.90, 2.95)**
Neighborhood socioeconomic status				
Lowest	**1.27 (1.02, 1.57)**	**1.94 (1.40, 2.69)**	**1.32 (1.15, 1.53)**	**1.83 (1.42, 2.36)**
Middle	1.07 (0.88, 1.31)	**1.51 (1.12, 2.04)**	**1.19 (1.05, 1.35)**	**1.55 (1.23, 1.96)**
Highest	Reference	Reference	Reference	Reference
Stage at diagnosis				
Local/regional	Reference	Reference	Reference	Reference
Distant	1.17 (0.69, 1.97)	**2.06 (1.13, 3.72)**	1.23 (0.85, 1.79)	**1.69 (1.01, 2.82)**
Initial treatment				
Total thyroidectomy and radioactive iodine	Reference	Reference	Reference	Reference
Total thyroidectomy without radioactive iodine	0.86 (0.72, 1.03)	1.03 (0.80, 1.33)	1.03 (0.92, 1.15)	0.90 (0.74, 1.10)
Partial thyroidectomy with radioactive iodine	0.80 (0.55, 1.17)	0.57 (0.29, 1.13)	0.89 (0.69, 1.14)	0.79 (0.50, 1.23)
Partial thyroidectomy without radioactive iodine	1.05 (0.81, 1.35)	1.17 (0.79, 1.74)	0.90 (0.75, 1.09)	1.06 (0.78, 1.44)
No thyroidectomy	**0.13 (0.02, 0.96)**	1.81 (0.76, 4.29)	0.64 (0.34, 1.19)	1.02 (0.44, 2.41)

*Note:* Bolded font indicates significant confidence interval at *p* < 0.05. Models controlled for year of diagnosis in 3‐year categories and state of residence. Moderate group has high incidence of diabetes and respiratory late effects, but no cardiovascular disease; Cardiovascular has high incidence of cardiovascular health events and nearly every other late effect.

Abbreviations: CI, confidence interval; OR, odds ratio.

Results from the Cox models were similar to the LCA model in terms of associations between age at diagnosis and greater hazards for the late effects included in the model (Table 4). However, the Cox model found lower hazards among females for CVD, liver, renal, and higher risk for respiratory late effects compared to males (Table 4). When compared to nH‐White survivors in the Cox proportional hazards model, we found higher hazards for diabetes, liver, and renal late effects among nH‐Black survivors; higher risk for diabetes and liver late effects among Hispanic survivors; and borderline higher risk for diabetes among Asian survivors.

**TABLE 4:** Multivariable‐adjusted hazard ratios and associated 95% confidence intervals for the increased risk of late effects among AYA thyroid cancer survivor cohort.

	Cardiovascular	Diabetes/Pancreatic	Liver	Renal	Respiratory	Secondary cancer
	HR (95% CIs)	HR (95% CIs)	HR (95% CIs)	HR (95% CIs)	HR (95% CIs)	HR (95% CIs)
Age at diagnosis, year						
15–19	Reference	Reference	Reference	Reference	Reference	Reference
20–29	**1.86 (1.11, 3.12)**	**2.58 (1.45, 4.58)**	1.16 (0.67, 2.02)	1.43 (0.59, 3.42)	1.16 (0.87, 1.54)	**2.96 (1.07, 8.17)**
30–39	**2.39 (1.44, 3.97)**	**4.42 (2.52, 7.78)**	**1.86 (1.09, 3.16)**	**2.41 (1.03, 5.59)**	1.18 (0.89, 1.57)	**5.27 (1.94, 14.28)**
Sex						
Female	**0.79 (0.63, 0.98)**	0.95 (0.76, 1.18)	**0.74 (0.56, 0.96)**	**0.59 (0.41, 0.86)**	**1.47 (1.22, 1.78)**	1.34 (0.96, 1.87)
Male	Reference	Reference	Reference	Reference	Reference	Reference
Race/ethnicity						
Non‐Hispanic White	Reference	Reference	Reference	Reference	Reference	Reference
Non‐Hispanic Black	1.28 (0.79, 2.08)	**2.30 (1.50, 3.54)**	**1.93 (1.08, 3.46)**	**2.46 (1.27, 4.76)**	1.22 (0.87, 1.69)	0.82 (0.37, 1.85)
Hispanic	0.98 (0.79, 1.22)	**2.23 (1.84, 2.72)**	**1.94 (1.49, 2.53)**	1.11 (0.75, 1.62)	0.93 (0.80, 1.08)	1.07 (0.81, 1.43)
Asian/Pacific Islander	0.72 (0.52, 0.98)	**1.32 (1.00, 1.74)**	0.96 (0.65, 1.42)	1.15 (0.70, 1.87)	0.59 (0.48, 0.74)	1.26 (0.91, 1.74)
American Indian	0.93 (0.50, 1.74)	1.36 (0.75, 2.44)	1.49 (0.73, 3.04)	0.97 (0.30, 3.14)	1.52 (1.06, 2.19)	1.29 (0.60, 2.76)
Health insurance						
Private/military	Reference	Reference	Reference	Reference	Reference	Reference
Public	**1.95 (1.57, 2.43)**	**1.97 (1.61, 2.41)**	**1.96 (1.49, 2.57)**	**3.26 (2.28, 4.65)**	**1.68 (1.43, 1.98)**	0.92 (0.63, 1.34)
Neighborhood socioeconomic status						
Lowest	**1.76 (1.38, 2.26)**	**1.57 (1.25, 1.98)**	1.12 (0.83, 1.52)	**1.63 (1.05, 2.51)**	**1.44 (1.21, 1.73)**	1.09 (0.80, 1.47)
Middle	**1.36 (1.09, 1.71)**	**1.25 (1.01, 1.55)**	1.05 (0.80, 1.38)	**1.55 (1.03, 2.33)**	**1.46 (1.26, 1.71)**	0.92 (0.70, 1.21)
Highest	Reference	Reference	Reference	Reference	Reference	Reference
Stage at diagnosis						
Local/regional	Reference	Reference	Reference	Reference	Reference	Reference
Distant	**2.05 (1.31, 3.23)**	1.15 (0.68, 1.96)	0.64 (0.26, 1.57)	1.03 (0.39, 2.73)	1.22 (0.80, 1.85)	1.35 (0.64, 2.86)
Initial treatment						
Total thyroidectomy and radioactive iodine	Reference	Reference	Reference	Reference	Reference	Reference
Total thyroidectomy without radioactive iodine	1.12 (0.93, 1.37)	0.86 (0.72, 1.03)	1.00 (0.78, 1.27)	1.29 (0.92, 1.82)	1.02 (0.89, 1.16)	0.93 (0.72, 1.21)
Partial thyroidectomy with radioactive iodine	0.97 (0.64, 1.46)	0.66 (0.43, 1.01)	1.12 (0.69, 1.82)	0.68 (0.27, 1.68)	0.90 (0.66, 1.22)	0.89 (0.51, 1.55)
Partial thyroidectomy without radioactive iodine	0.99 (0.72, 1.36)	1.06 (0.82, 1.38)	1.34 (0.96, 1.89)	1.28 (0.76, 2.16)	0.90 (0.72, 1.14)	0.94 (0.63, 1.42)
No thyroidectomy	1.89 (0.91, 3.93)	0.64 (0.24, 1.72)	0.34 (0.05, 2.43)	2.06 (0.64, 6.64)	0.60 (0.27, 1.34)	0.73 (0.18, 2.96)

*Note:* Bolded font indicates significant confidence interval at *p* < 0.05. Models controlled for year of diagnosis in 3‐year categories and state of residence.

Abbreviations: CI, confidence interval; HR, hazard ratio.

Relative to survivors treated with total thyroidectomy and radioactive iodine, we did not find differences in the risk for late effects for other combinations of thyroidectomy and radioactive iodine/no radioactive iodine treatment in the summed number model or Cox proportional hazards models. In the model using the LCA classes as the outcome, we found a reduction of odds for being in the Moderate Disease class among survivors treated with no thyroidectomy relative to survivors treated with total thyroidectomy and radioactive iodine.

## 4 Discussion

In this analysis of a large cohort of AYA thyroid cancer survivors from two states, we report that a majority of survivors have a low risk for multiple late effects up to 15 years after their diagnosis, which is supported by the LCA. According to the short‐term LCA, an estimated 1% of AYA thyroid cancer survivors may have cardiovascular and renal disease, and an estimated 11% may have diabetes, liver, and respiratory disease (Moderate) 5 years after their thyroid cancer diagnosis (3 years after cohort entry). Over the entire study period, the long‐term LCA estimates that 3% of AYA thyroid survivors were characterized by cardiovascular late effects along with other conditions, and 9% had diabetes, liver, and respiratory late effects. Our findings suggest that the risk for late effects among thyroid cancer survivors may be driven by a small proportion of survivors with many late effects rather than just a single late effect [4].

Notably, our analyses found that survivors who were non‐Hispanic Black and Hispanic, had public insurance, resided in lower SES neighborhoods, or were diagnosed at distant stage experienced greater odds of being in the Cardiovascular class. This supports prior analyses in California finding increased incidence of medical conditions in non‐Hispanic Black and Hispanic cancer survivors [14]. Latent class analysis is becoming a more utilized method to understand patterns in morbidity [22]. While the results from separate models using the LCA classes, summed number, and Cox proportional hazards methods were somewhat similar, the LCA provided an additional benefit by identifying the types of late effects that are most commonly found in the Moderate or Cardiovascular classes. The results from the short‐term LCA also support the potential co‐occurrence of cardiovascular disease and renal disease for some survivors. Thus, analyses summing late effects or considering late effects independently could potentially miss important disease clusters that may require more coordinated care approaches for multiple linked chronic conditions.

A small percentage of the survivors in the Cardiovascular class from the long‐term LCA were in the Low or Moderate class in the short‐term LCA. While this finding is largely explained by the differences in the use of cardiovascular late effects to classify survivors in the long‐term and short‐term LCA models, we also found that survivors in the Low/Moderate short‐term LCA class and Cardiovascular long‐term LCA class had public health insurance and lower neighborhood socioeconomic status [14]. The association between public insurance at diagnosis and higher risk of late effects could be related to the demographics of populations that are eligible to be enrolled in public insurance programs (e.g., low income or individuals with disabilities). Additionally, the regulatory complexity of maintaining public health insurance could result in the discontinuity of health insurance coverage and affect access to preventive care after treatment ends. Consistent with our prior findings in California [14], we also found that Non‐Hispanic Black and Hispanic survivors were more likely to be in the Moderate class than the Low class compared to non‐Hispanic White survivors; and those diagnosed with distant stage disease were more likely to be in the Cardiovascular class. These populations tend to have lower incomes than Non‐Hispanic White survivors and may also have higher preexisting obesity rates [25], which are associated with the late effects reported in this study.

Thyroid hormone is responsible for regulating a wide variety of physiologic processes and affects nearly every organ in the body [26]. Our study suggests that the risk for late effects does not differ among patients treated with or without thyroidectomy or radioactive iodine therapy. We found reduced odds for being in the Moderate class among survivors treated without thyroidectomy. This could be caused by greater medical surveillance due to the “watchful waiting approach” for low‐risk thyroid cancer or cancer treatments used to treat thyroid cancer without surgical intervention like radiofrequency ablation [27]. Treatment with chemotherapy is rare unless patients are diagnosed with metastatic disease [3, 28, 29]. Specifically, anthracycline, platinum‐based therapies, and taxanes can be used to treat thyroid cancer [30]. Consistent with our findings that AYA thyroid cancer survivors with distant disease were more likely to be in the Cardiovascular class, these chemotherapies are linked to long‐term increases in risk for cardiovascular disease [31]. As the number of survivors given chemotherapy was too small to analyze as a separate category and we do not have data on specific chemotherapy agents administered or on prescriptions to mitigate cardiotoxicity [31], these results warrant further investigation.

### 4.1 Limitations and Strengths

The maximum follow‐up for the cohort was 14 years from diagnosis, but the median was 7 years, which may be too short for certain late effects to emerge. While this multistate cohort provided a large sample size for analysis, our reliance on emergency department, hospitalization, and ambulatory surgery records may provide an underestimate of late effects that are diagnosed and treated in outpatient settings only, as well as biasing the detection of late effects to participants or conditions that require care in these settings. This largely allows us to track only severe outcomes that require this type of care. If we had outpatient or primary care data, the prevalence of diseases like diabetes likely would increase with data that covers more routine healthcare visits, so future studies should incorporate outpatient data.

Late effects were generally rare in this cohort, which created a small population of diseased survivors that provided data for the LCA. As two of the LCA classes contained less than 10% of the population, these two late effects classes could have been collapsed into a single group if we were to follow general guidance surrounding the LCA method [22]. However, the model AICs supported the use of the three classes, and the Moderate and Cardiovascular classes were divergent enough to keep them as separate groups. The length of follow‐up time was a potential source of concern, as participants who did not have as many years of follow‐up might not develop disease, which would reduce disease prevalence. To overcome this, we conducted the short‐term and long‐term LCA, which found generally similar late effects class patterns, although with some variation, likely due to differences in the emergence of late effects over follow‐up time. Additionally, we compared our findings to Cox proportional hazards models.

We also were unable to determine which late effects are associated with treatment due to small numbers. For example, second cancers are more likely associated with radioactive iodine treatment [32]. In contrast, cardiovascular disease may be associated with both thyroid removal and chemotherapy, depending on the agent used [30]. In addition, we did not have access to data on obesity prior to cancer diagnosis, and we were unable to identify comorbidities or health behaviors present after diagnosis (e.g., smoking) that may be associated with late effects. Last, we are focused on identifying novel associations and utilizing novel statistical methods rather than causal associations between treatment and late effects. The differences in disease incidence between populations with and without cancer could be better established if state‐based, matched comparison cohorts were available for the California data; the Utah data does have an age‐sex matched cohort, but this was not the focus of the current study.

Despite these limitations, our study provides novel insights into the prevalence of late effects among thyroid cancer survivors and which late effects may cluster together. We also used a large multi‐state cohort that provides demographic and geographic variability to examine late effects in this understudied population. Results from this cohort are not generalizable to the entire nation but do represent the composition of many Western states in the United States. This sample combined data from Utah and California, which provided a larger population of Hispanic survivors (31.7%) as well as Asian and Pacific Islander survivors (14.3%). The aggregation of Asian and Pacific Islander populations was done due to the limitations of data collection practices in the past but is problematic as socioeconomic risk factors for disease vary widely in these populations. The data also have very small percentages of Black and American Indian populations who are underrepresented in cancer survivorship research. Data from other regions of the United States can assist with increasing information about late effects in these underrepresented populations.

There are no indications that immortal person‐time bias was introduced by specifying a follow‐up period that begins two years after the first primary diagnosis. Our inclusion criteria were that survivors must have been alive beginning two years after diagnosis. In addition, we only examined new conditions that occurred after the study start date. Thus, no time in which the event could not have occurred was included in the analysis.

## 5 Conclusion

A small population of AYA thyroid cancer survivors appears to be at risk for many co‐morbid late effects. Our results support the clustering of certain diseases together, notably cardiovascular and renal disease within 5 years of diagnosis. Distant stage at diagnosis, non‐Hispanic Black or Hispanic race/ethnicity, low neighborhood SES, and public insurance were associated with higher odds of being in the Moderate or Cardiovascular classes in the long term. Thus, multidisciplinary survivorship care may be necessary for a small but potentially high disease burden of younger thyroid cancer survivors experiencing multiple late effects.

## Author Contributions

**Judy Y. Ou:** conceptualization (lead), formal analysis (supporting), investigation (equal), methodology (lead), validation (supporting), writing – original draft (lead), writing – review and editing (lead). **Theresa Keegan:** data curation (supporting), formal analysis (supporting), methodology (supporting), resources (equal), supervision (equal), writing – review and editing (supporting). **Qian W. Li:** formal analysis (supporting), writing – review and editing (supporting). **Heydon K. Kaddas:** project administration (supporting), writing – original draft (supporting), writing – review and editing (supporting). **Renata Abrahão:** writing – original draft (supporting), writing – review and editing (supporting). **Ann Brunson:** methodology (supporting), supervision (supporting), visualization (equal), writing – original draft (supporting), writing – review and editing (supporting). **Candice A. M. Sauder:** writing – review and editing (supporting). **Jessica Chubak:** writing – review and editing (supporting). **Lawrence H. Kushi:** resources (lead), writing – review and editing (supporting). **Erin E. Hahn:** resources (supporting), writing – review and editing (supporting). **Chun Chao:** resources (supporting), writing – review and editing (supporting). **Hazel B. Nichols:** resources (supporting), writing – review and editing (supporting). **Anne C. Kirchhoff:** data curation (lead), investigation (lead), project administration (lead), writing – original draft (supporting), writing – review and editing (supporting).

## Ethics Statement

This study was approved by Institutional Review Boards at the University of Utah, the Utah Resource for Genetic and Epidemiologic Research, the California Committee for the Protection of Human Subjects, and Kaiser Permanente Northern California Institutional Review Board.

## Conflicts of Interest

The authors declare no conflicts of interest.

## Code Availability Statement

The ICD9 and 10 codes used in this study are available from the corresponding author upon reasonable request.

## Supporting information


**Data S1:** Supporting Information.

## Data Availability

The data that support the findings of this study are available from California Cancer Registry (CCR) and the Utah Population Database/Utah Cancer Registry. Restrictions apply to the availability of these data, which were used under license for this study. Data are available from the authors with the permission of Institutional Review Boards at the University of Utah, the Utah Resource for Genetic and Epidemiologic Research, the California Committee for the Protection of Human Subjects, and Kaiser Permanente Northern California Institutional Review Board.
