# Supplementary material for: Identifying Patterns of Late Effects With Latent Class Analysis Among Adolescent and Young Adult Thyroid Cancer Survivors in California and Utah
Source: Cancer Med. 2025 Nov 27;14(23):e71316. doi: 10.1002/cam4.71316 (PMC12659762; doi:10.1002/cam4.71316)
Supplement: Supplementary file 1 — Data S1: Supporting Information. [file CAM4-14-e71316-s001.docx]

Supplemental Table 1. Risk factors associated with group classification identified in the short-term latent class analysis using multinomial logistic regression among AYA thyroid cancer survivor cohort

|  | Moderate | Cardiovascular/Renal |
| --- | --- | --- |
|  | N = 1030 (7.22%) | N = 79 (0.55%) |
|  | OR (95% CI) | OR (95% CI) |
| **Age at diagnosis, year** |  |  |
| 15-19 | Reference | Reference |
| 20-29 | 1.56 (1.08,2.24) | 1.96 (0.45,8.60) |
| 30-39 | 2.45 (1.72,3.49) | 4.36 (1.05,18.2) |
| **Sex** |  |  |
| Female | 0.85 (0.72,1.01) | 0.63 (0.37,1.08) |
| Male | Reference | Reference |
| **Race/ethnicity** |  |  |
| Non-Hispanic White | Reference | Reference |
| Non-Hispanic Black | 1.42 (0.98,2.05) | 3.21 (1.42,7.26) |
| Hispanic | 1.61 (1.38,1.89) | 0.97 (0.55,1.70) |
| Asian/Pacific Islander | 0.95 (0.77,1.17) | 1.17 (0.60,2.26) |
| American Indian | 1.14 (0.69,1.87) | 1.47 (0.34,6.30) |
| **Health insurance** |  |  |
| Private/military | Reference | Reference |
| Public | 1.83 (1.55,2.16) | 4.06 (2.46,6.68) |
| **Neighborhood socioeconomic status** |  |  |
| Lowest | 1.45 (1.21,1.74) | 1.97 (1.03,3.76) |
| Middle | 1.21 (1.03,1.43) | 1.66 (0.91,3.02) |
| Highest | Reference | Reference |
| **Stage at diagnosis** |  |  |
| Local/regional | Reference | Reference |
| Distant | 1.77 (1.22,2.58) | 1.16 (0.28,4.90) |
| **Initial treatment** |  |  |
| Total thyroidectomy and radioactive iodine | **Reference** | **Reference** |
| Total thyroidectomy without radioactive iodine | 0.94 (0.81,1.08) | 1.16 (0.71,1.88) |
| Partial thyroidectomy with radioactive iodine | 0.89 (0.64,1.23) | 0.59 (0.14,2.46) |
| Partial thyroidectomy without radioactive iodine | 1.00 (0.79,1.26) | 1.22 (0.56,2.65) |
| No thyroidectomy | 0.87 (0.45,1.69) | 1.00 (0.13,7.68) |
| Abbreviations: OR=Odds ratio, CI=Confidence Interval  Bolded font indicates significant confidence interval at p<0.05  Models controlled for year of diagnosis in 3-year categories and state of residence.  Moderate group has high incidence of diabetes and respiratory late effects, but no cardiovascular disease  Reference group is low | | |
|  | | |
